# Succession of pit mud archaeal communities and metabolic networks: the hidden key to the unique flavor of strong-flavor Baijiu

**DOI:** 10.1128/aem.00099-26

**Published:** 2026-04-20

**Authors:** Qilin Huang, Ying Liu, Lei Tian, Xianping Qiu, Yanzhong Li, Yu Li, Yinru Liu, Shiyu Yang, Tongwei Guan

**Affiliations:** 1 College of Food and Biological Engineering, Xihua University, Food Microbiology Key Laboratory of Sichuan Province 691451, Chengdu, People's Republic of China; 2 Sichuan Quanxing Distillery Co., Ltd., Chengdu, People's Republic of China; 3 Sichuan Jiangkouchun Longding Distillery Co., Ltd, Bazhong, People's Republic of China; 4 Sichuan Tujiu Distillery Co., Ltd, Nanchong, People's Republic of China; University of Nebraska-Lincoln, Lincoln, Nebraska, USA

**Keywords:** pit mud, archaeal community, strong-flavor Baijiu, flavor compounds

## Abstract

The microbial community in pit mud, particularly the archaeal community, plays a pivotal role in the solid-state multi-microorganism fermentation process of strong-flavor Baijiu. This study systematically analyzed physicochemical properties, archaeal succession, and volatile metabolites across pit ages (3, 45, 100 years) and spatial layers (upper/middle/lower) in pit mud. It revealed the mechanisms by which environmental factors drive archaeal community composition and metabolic function. Results revealed marked spatiotemporal variations in pit mud’s archaea and metabolites, with 45-year-old samples (especially mid-layer) showing peak archaeal diversity and highest volatile compound richness (predominantly esters). The phyla *Methanobacteriota* and *Halobacteriota* were identified as the dominant archaeal groups, with the genus *Methanothrix* being exclusively detected in 100-year pit mud. The genus *Methanoculleus* reached its highest abundance in 45-year pit mud and exhibited a progressive increase along the pit depth gradient. The Pearson correlation analysis revealed significant associations between the archaeal community structure and key environmental factors, including pH, humus, ammonium nitrogen, available phosphorus, and multiple metal elements, suggesting that these factors collectively influence archaeal community assembly. The functional prediction (PICRUSt2) further elucidated the synergistic relationship between archaeal key enzymes involved in methanogenesis and critical flavor compound synthesis. This study offers a new perspective on the notion that “older pit mud is always better.” Our findings show that, in this evaluation, 45-year-old pit mud exhibited superior overall quality compared to 100-year-old pit mud. This indicates that pit mud quality is closely related to archaeal succession and influenced by multiple factors.

**IMPORTANCE:**

The assembly of microbial communities is a long-term process, and temporal patterns in environmental communities cannot be studied in isolation—spatial patterns must also be considered. The functional roles of rare archaeal communities in the pit mud ecosystem are equally important, yet they remain understudied. To elucidate the temporal and spatial dynamics of archaeal succession in this ecosystem, the archaeal communities in pit mud from different temporal and spatial contexts were investigated. This study offers a new perspective on the notion that “older pit mud is always better.” Our findings show that, in this evaluation, 45-year-old pit mud exhibited superior overall quality compared to 100-year-old pit mud, suggesting that pit mud quality is likely influenced by multiple complex factors.

## INTRODUCTION

Baijiu is a traditional Chinese distilled liquor and an alcoholic beverage with distinctive national characteristics (1, 2). Due to distinct flavor profiles, Baijiu is categorized into 12 aroma types, with strong flavor, light flavor, and sauce flavor recognized as the three fundamental aroma styles (3). Chinese strong-flavor Baijiu (CSFB) is produced through the anaerobic fermentation of crushed and mixed grains (sorghum, wheat, rice, and corn) with Daqu starter culture in pit mud (PM)-lined cellars (4, 5). The distilled CSFB is characterized by intense pit aroma, crisp sweetness, harmonious fragrance, and a lingering clean finish, making it highly popular among consumers (6).

Microbial communities play pivotal roles across ecosystems of all scales, from driving global biogeochemical cycles to facilitating household food fermentation (7). Pit mud is a fermented clay medium harboring diverse anaerobic microorganisms, which adheres to the bottom and inner walls of fermentation pits (8). The microbial interactions and metabolic activities within pit mud play pivotal roles in liquor fermentation. Fundamentally, the quality of pit mud is determined by the anaerobic microbial communities colonizing it (8). In pit mud ecosystems, prokaryotes (predominantly bacteria with a minor archaeal component) constitute the dominant microbial populations (9–11). The bacterial community is predominantly composed of obligate or facultative anaerobes, with *Clostridium* species being the most abundant. These bacteria are key producers of metabolic compounds such as butyric acid and hexanoic acid (10, 11). These organic acids undergo esterification with ethanol to form ethyl hexanoate, ethyl butyrate, and other esters, which impart the distinctive pit mud aroma to Baijiu (12). Archaea are prokaryotes ubiquitously distributed in extreme environments (e.g., submarine hydrothermal vents, hot springs, hypersaline lakes with saturated salt concentrations, and strict anaerobic habitats) as well as animal bodies (12, 13). Archaea represent a crucial functional microbial group in pit mud. Research by Ren et al. on premium-quality pit mud revealed that its archaeal community is predominantly composed of methanogenic genera (14). A comparative study by Cai et al. of degraded and normal pit mud revealed that archaeal communities were the primary differentiating factor. Specifically, the degraded pit mud showed significant enrichment of *Methanobacterium* and *Methanothermobacter* genera compared to normal pit mud (15). Furthermore, Zhang et al. demonstrated that inoculating pit mud with methanogenic consortia not only reduced methanol content in the liquor but also enhanced the production of organic acids (hexanoic acid, acetic acid, and butyric acid), thereby improving the aromatic profile of Baijiu (16). Currently, the relatively abundant pit mud bacteria have been more extensively studied, while the functional importance of rare archaeal communities in the pit mud ecosystem remains under-researched (17). Thus, investigating archaea in pit mud holds significant implications for both liquor quality enhancement and fermentation pit management.

Pit age, spatial location, physicochemical properties, and environmental conditions are all critical factors governing the microbial community composition and quality of pit mud (5). A study by Xia et al. on microbial succession drivers in strong-flavor Baijiu pit mud revealed that NH_4_^+^-N, moisture content, and available phosphorus were the primary determinants of bacterial communities, with older pit mud exhibiting enhanced butyric acid synthesis potential (18). A study on pit mud variations across distilleries in the Yibin production region revealed significant geographical divergence, with distinct bacterial and archaeal communities driving differential volatile compound profiles (19). The formation of microbial communities is a long-term process. The temporal patterns in the environmental community cannot be studied in isolation; spatial patterns also need to be considered, and microorganisms at different cellar ages and in different spatial locations are in a dynamic succession process (20). The community structure of the pit mud is closely related to the age of the pit. Exploration of prokaryotes in 40-year-old and 400-year-old pit mud revealed that the abundance of the main bacteria *Caproiciproducens* in mature pit mud increases with the increase of the age of the pit (21). The study on pit mud aged 5, 30, and 100 years revealed that the abundance of 7 core genera of *Firmicutes* phylum microorganisms increased with pit age (21). Additionally, practical production has demonstrated that the quality of raw liquor distilled from fermented grains varies across different layers (22). The exploration of pit mud with different fermentation ages and spatial positions can contribute to both pit mud quality control and the improvement of raw liquor quality (22).

Therefore, to elucidate the succession patterns of archaea in pit mud across different fermentation ages and spatial positions, this study investigated the archaeal community composition and physicochemical characteristic variations in pit mud aged 3, 45, and 100 years. Additionally, the functional roles of archaeal community structures in pit mud and their impact on flavor compounds were systematically elucidated. These findings facilitate a deeper understanding of the functional roles of archaea in pit mud, as well as their relationships with physicochemical factors and flavor compounds, thereby providing theoretical guidance for enhancing and reconstructing microbial communities in newly constructed pit mud.

## MATERIALS AND METHODS

### Sample collection

Samples of pit mud aged 3, 45, and 100 years were collected from a distillery in Sichuan Province. For each age group, three cellars of the same age were randomly selected as biological replicates. Samples were taken from the upper (30 cm below the cellar opening), middle (1.5 m below the opening), and lower (near the cellar bottom) layers of each cellar. Samples were collected from three depths (upper, middle, and lower) of three parallel cellars in each age group. Approximately 5 cm of surface pit mud was excavated from each layer, and 50 g was taken, mixed uniformly, and divided into three portions for testing (resulting in a total of 27 samples).

The collected pit mud samples were immediately transferred into sterile sampling bags and transported to the laboratory. Based on temporal and spatial differences, the samples were labeled as follows: 100-year-old pit mud: upper (CT), middle (CM), and lower (CD); 45-year-old pit mud: upper (BT), middle (BM), and lower (BD); 3-year-old pit mud: upper (AT), middle (AM), and lower (AD).

### Physicochemical properties of pit mud

The moisture content of pit mud was determined using the dry/wet weight method (dried at 105°C for 6 hours), followed by pH measurement with a pH meter. The ammonium fluoride-hydrochloric acid colorimetric method (referenced to DB34/T 2266-2014) was employed to measure the AP content. The potassium dichromate oxidation method was used to determine the NH₄^+^-N content. The measurement was conducted using a spectrophotometer at a wavelength of 425 nm, based on a colorimetric principle involving nano-reagents (23). The humus content was determined using the Nessler’s reagent colorimetric method (referenced to DB34/T 2265-2014).

### Metal elements in pit mud

The pit mud samples (3, 45, and 100 years) were dried at 60°C for 4 h, pulverized, and sieved through a 100-mesh sieve. Accurately weigh 1 g of each pit mud sample into a microwave digestion vessel, then add 7.0 mL of HNO_3_ and 0.5 mL of H_2_O_2_. After standing for 3 h, perform microwave digestion. Following cooling, open the vessel to release pressure, rinse the inner lid with deionized water, and heat the digestion tube on an electric hotplate at 150°C for 60 min. Finally, dilute the digestate to 50 mL with 1% HNO_3_ for subsequent analysis (24).

Metal ion concentrations were determined using inductively coupled plasma mass spectrometry (ICP-MS; Model: NexION 350X, PerkinElmer, USA) (25). Instrumental parameters for ICP-MS analysis: RF power: 1.55 kW; sampling depth: 8 mm; plasma gas flow rate: 15 L/min; auxiliary gas/sheath gas/dilution gas flow rates: 1 L/min (each); spray chamber temperature: 2°C; peristaltic pump speed: 0.1 revolutions per second.

### Pit mud analysis by comprehensive two-dimensional gas chromatography-mass spectrometry

The analysis was performed according to previously reported methods using comprehensive two-dimensional gas chromatography-mass spectrometry (GC×GC-MS; GC-2010, Shimadzu, Japan) with a DB-FFAP capillary column (60 m × 0.25 mm × 0.25 µm) for volatile compound detection (26). Accurately add 1 g of pit mud and 20 μL internal standard (2-octanol, 0.822 mg/mL in ethanol) into a 20 mL headspace vial, followed by 8 mL salt solution (0.85% NaCl + 1% CaCl_2_) and 2.5 g NaCl. The column carrier gas was high-purity helium at a 1 mL/min flow rate. The column oven temperature was programmed at 40°C (hold 5 min), then ramped at 5°C/min to 230°C, with mass spectra acquired in electron ionization mode at 70 eV using a scan range of m/z 25–550 amu. The ion source temperature was maintained at 230°C. Volatile compounds were identified and quantified following previously established methodologies (27).

### High-throughput sequencing of archaea in pit mud

Total microbial genomic DNA was extracted from all 27 samples using the E.Z.N.A. Soil DNA Kit (Omega Bio-tek, Norcross, GA, USA) following the manufacturer’s instructions. The quality and concentration of the DNA were determined by 1.0% agarose gel electrophoresis and a NanoDrop 2000 spectrophotometer (Thermo Scientific, United States), and the samples were stored at −80°C for further use. The V4–V5 region of archaea was amplified by PCR using the BIO-RAD t100 Thermal Cycler (USA) with the primers 524F10extF (5′-TGYCAGCCGCCGCGGTAA-3′) and Arch958RmodR (5′-YCCGGCGTTGAVTCCAATT-3′) (28). The amplified products were separated by 2% agarose gel electrophoresis, purified, and quantified, followed by sequencing on the Illumina NextSeq 2000 platform (Shanghai Meiji Biomedical Technology Co., Ltd.).

### Statistical analysis

Duncan’s test and analysis of variance were conducted using SPSS 27.0 software for significance testing (*P* < 0.05). Line charts and bar charts were plotted using Origin 2024 software. Orthogonal partial least squares-discriminant analysis (OPLS-DA) was performed using SIMCA-P 14.1 software (Umetrics, Sweden). Functional composition analysis of the archaeal community in PM was conducted using PICRUSt2.

## RESULTS AND DISCUSSION

### Changes in physical and chemical factors of pit mud in different spaces and years

**Fig 1:**
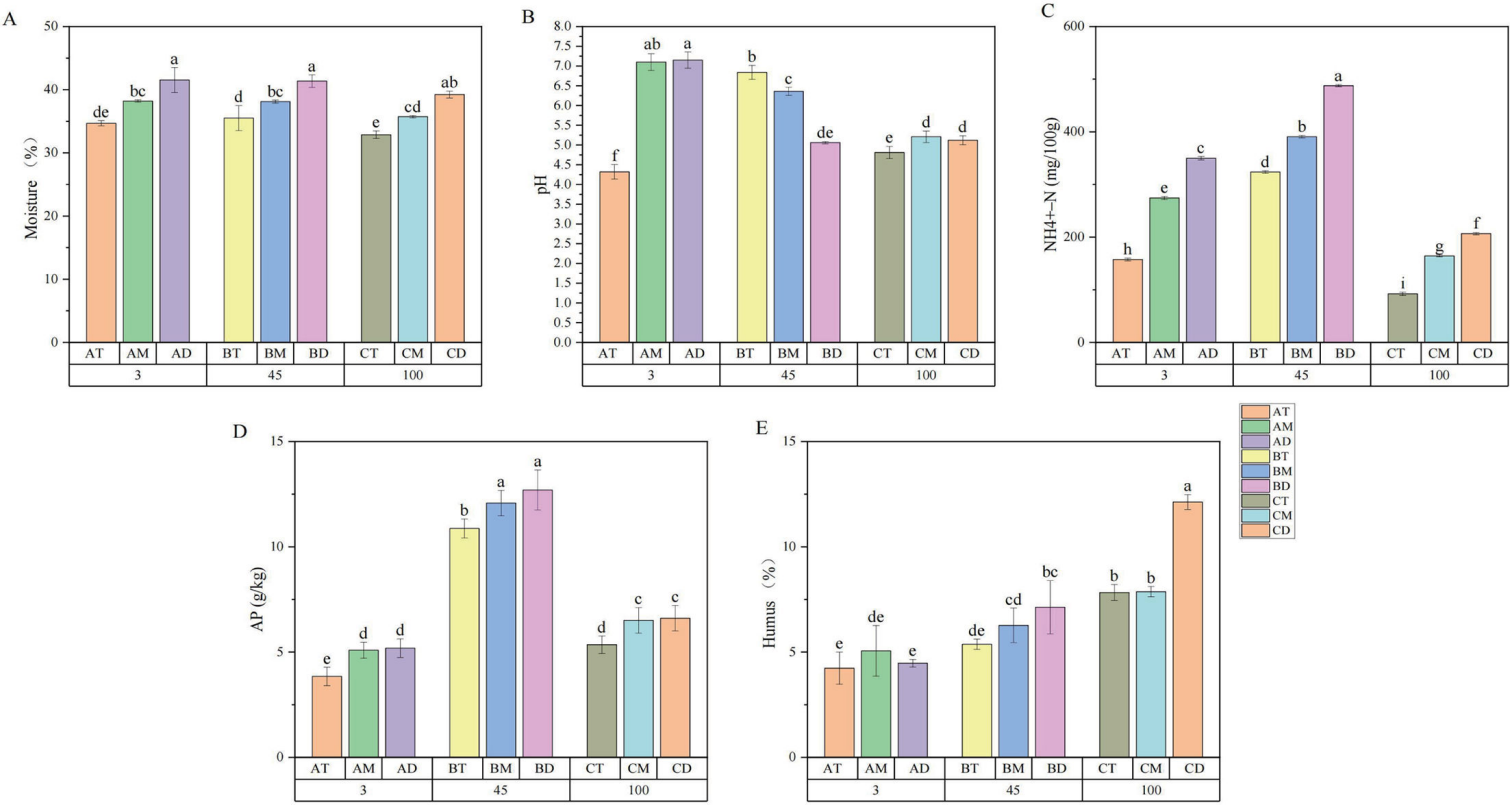
Physicochemical properties of pit mud across different ages and spatial locations. (**A**) Moisture content, (**B**) pH, (**C**) NH₄^+^-N (ammonium nitrogen), (**D**) AP (available phosphorus), and (**E**) humus. Results are presented as the mean ± SD of three independent experiments. Different lowercase letters indicate significant differences between samples (*P* < 0.05).

The moisture content, pH, NH₄^+^-N, AP, and humus levels of pit mud samples from different locations and years were compared (Fig. 1A through E). Pit mud moisture plays a crucial role in organic matter dissolution, nutrient transport, and microbial metabolism (29). Studies indicate that high-quality pit mud should maintain a moisture content of approximately 40%, typically ranging between 38% and 44% (30). Among pits of different ages at the same location, significant differences in moisture content were observed between the upper layers of 3-/45-year pits and 100-year pits, with the 100-year upper layer showing the lowest moisture level. The increase in water content with pit depth is likely attributed to the downward seepage of yellow liquid (a byproduct of grain fermentation) and reduced evaporation capacity at the bottom layers (31). NH_4_^+^-N is the essential nitrogen source for the survival of microorganisms in the pit mud. It reaches its maximum level in the 45-year-old cellar and increases with the increase of cellar depth (19). Phosphorus is a vital component of microbial cells, and cellular decomposition leads to increased phosphorus content (32). Within pits of the same age, significant differences in AP content were observed between the upper layer and both middle/lower layers, while no significant variation existed between middle and lower layers. The variation trends of AP and NH₄^+^-N were consistent across different pit ages, both peaking at 45 years. Studies indicate that moisture, NH₄^+^-N, and AP often show significant correlations with pit age or pit mud quality (18). An appropriate pH not only promotes microbial growth but also facilitates the formation of aroma compounds in strong-flavor Baijiu (18). Overall, pH tends to decrease with increasing pit age. Previous studies have shown that in different production regions, high-quality pit mud generally exhibits a pH range of 5.0–7.0. A pH value closer to 5.0 indicates better pit mud quality (30, 33). Humus, a microbially enriched medium, functions as a natural buffer and ion exchanger that enhances water retention while inhibiting calcium/iron lactate formation, thereby preventing pit mud hardening and degradation (34). Humus content increases with both pit age and depth within individual fermentation pits.

Based on the comprehensive results, both 45- and 100-year pit mud significantly outperform 3-year pit mud in multiple key physicochemical indicators, reflecting the regulatory role of pit mud age on its microenvironment. The 45-year-old pit mud shows significant advantages in physicochemical indicators; however, the quality differences between 45- and 100-year-old pit mud require further analysis through volatile metabolites and microbial community profiling.

### Differences in metal elements of pit mud at different spatial levels

**Fig 2:**
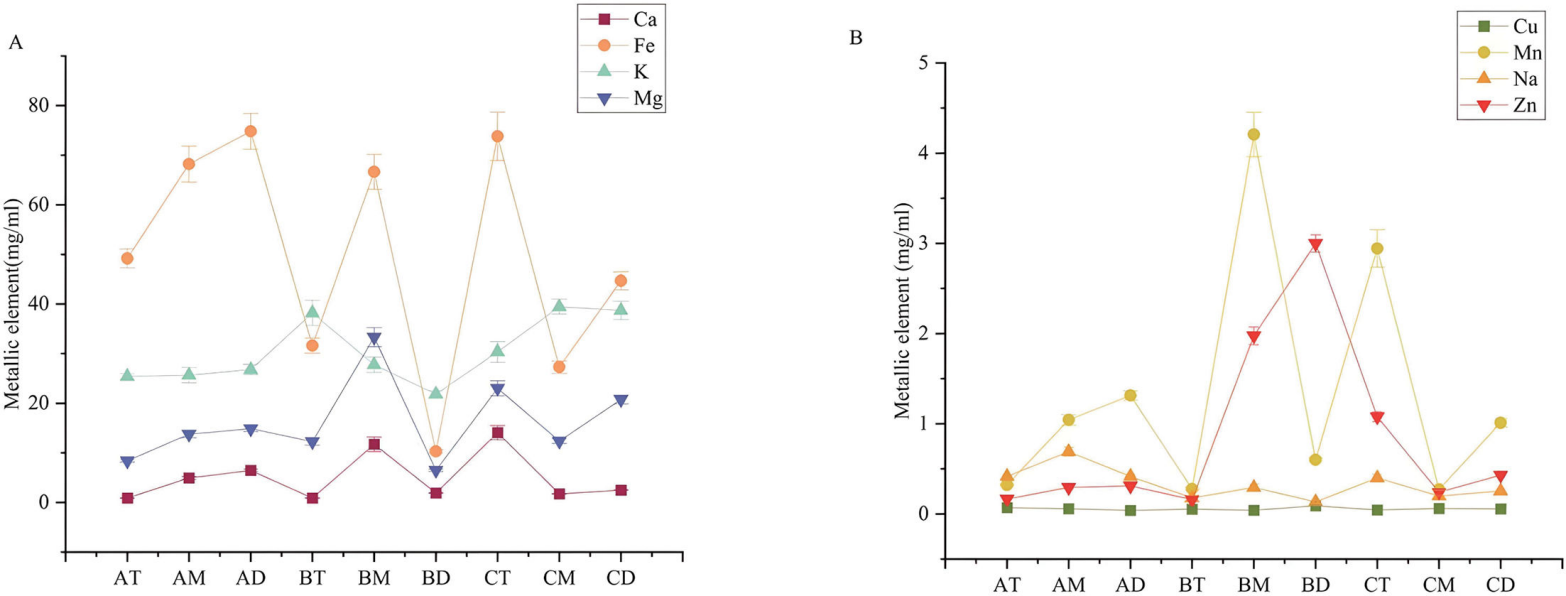
Metal element concentrations in pit mud across different ages and spatial layers. (**A**) Ca, Fe, K, and Mg. (**B**) Cu, Mn, Na, and Zn.

Pit mud constitutes a complex micro-ecosystem where metal elements (Ca, Mg, Zn, Cu, Fe, and Mn) not only enhance the biosynthesis and accumulation of flavor compounds during fermentation but also facilitate the formation of trace element complexes（Fig. 2A and B) (35). The calcium in the pit mud mainly comes from the degradation of grains, the water used for brewing, and the degradation of calcium carbonate in the yellow silt used as the raw material for the cellar mud (36). The spatial distribution of calcium (Ca) in pit mud exhibits age-dependent patterns: maximum Ca content occurs in the bottom layer of 3-year pits, shifts to the middle layer in 45-year pits, and ultimately concentrates in the upper layer of centennial (100 years) pits, demonstrating a progressive upward migration of Ca accumulation with pit aging. The relatively high copper content in BD pit mud is considered one of the key reasons for its prominent ammonium-nitrogen content. Copper is involved in the activity of enzymes such as urease and polyphenol oxidase, which enhances the bioavailability of nutrients in the pit mud (36). In the BD sample, the copper content is the highest. Due to the influence of factors such as sour mash soaking and pH, ferrous lactate accumulates and crystallizes, which causes toxic effects on the microorganisms in the cellar mud, making the cellar mud lose its aroma-producing function and eventually aging. Therefore, the Fe content in high-quality cellar mud is lower than that in new cellar mud and aged cellar mud (37). With increasing pit age, oxygen depletion creates reducing conditions that enhance iron solubility but reduce total iron content over time (38). Furthermore, Fe³^+^ modulates ester content in Baijiu through free metal-ion catalyzed reactions (39). BD showed the lowest K, Mg, and Na but the highest Zn levels. K supplementation boosts alkaline phosphatase activity, enhancing soil phosphorus bioavailability (40). In summary, the content, distribution, and speciation transformation of various metal elements in pit mud are closely related to pit age and the physicochemical state of the mud. Together, they influence the formation of flavor compounds in strong-flavor Baijiu and the quality of the pit mud.

### GC×GC-MS analysis of volatile compounds and identification of key aroma substances

#### Analysis of volatile compounds in pit mud across different age layers

**Fig 3:**
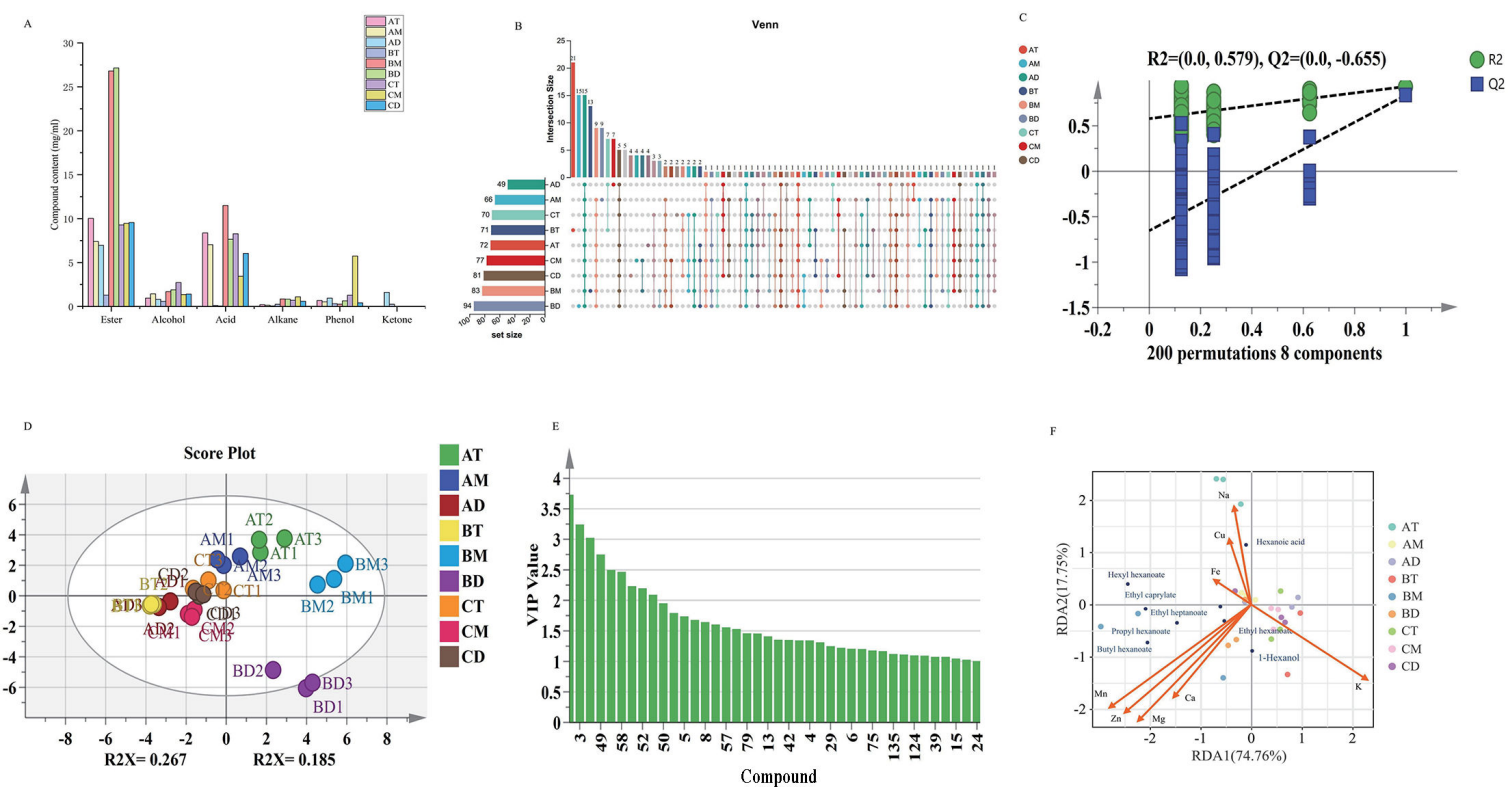
(**A**) Categories and concentrations of compounds in pit mud. (**B**) Upset Venn diagram of volatile compounds in pit mud, showing the shared and unique volatile compounds among different pit mud sample groups. (**C**) Permutation test plot for the OPLS-DA model (200 iterations). (**D**) PLS-DA score plot of the volatile compounds, showing the overall separation and clustering of pit mud samples based on their metabolic profiles. (**E**) Compounds with variable importance in projection (VIP) >1. (**F**) Redundancy analysis (RDA) between key differential compounds and metal elements.

The microorganisms in the cellar mud can migrate to the fermented grains and produce flavor substances. After distillation, these substances enter the liquor and affect its quality and flavor. Therefore, the study of the volatile components in the cellar mud is of great significance (8). The results indicated that the compounds in pit mud were primarily categorized into esters, alcohols, acids, ketones, phenols, and alkanes (Fig. 3A), with trace amounts of other compounds such as aldehydes and pyrazines also present. Figure 3A through B reveals that BD contained the most abundant compounds (42.69 mg/mL), closely followed by BM (42.61 mg/mL), with both significantly exceeding other pit muds, indicating the 45-year fermentation pit creates optimal conditions for functional microbiota (41). They are usually formed by the esterification of short-chain acids and alcohols. Among them, the content of BD esters is the highest, reaching 27.16 mg/mL, accounting for more than 50% of the total compounds. The second is BM (26.80 mg/mL) (42). The observed increase in ester diversity with pit depth in both 45-year and centennial (100-year) pit mud systems demonstrates enhanced esterification potential within the deep-layer microbial communities of mature fermentation ecosystems.

Ethyl hexanoate has a strong fruity aroma and is an important aroma component in the production of liquor. Microbial metabolism is its main source, mainly consisting of the yeasts, molds, and bacteria that produce aroma in the cellar mud (43–45). Over a period of 3 years, the content of ethyl hexanoate decreased as the depth of the cellar increased, which was contrary to the trend observed in a 100-year-old cellar. The reason might be that the upper layer of the new cellar has the highest oxygen content, and most of the functional microorganisms are aerobic bacteria. Meanwhile, the microorganisms in the old cellar tend to be stable. Sample BM exhibited the highest ethyl hexanoate concentration (7.40 mg/mL), surpassing other pit mud samples by two- to fourfold, followed by BD (3.60 mg/mL). Studies have shown that the production of ethyl caproate is related to the *Clostridium* genus, suggesting that the abundance of *Clostridium* in the BM may be relatively high (44–46).

Acids modulate Baijiu flavor by neutralizing harsh spiciness and bitterness while enhancing sweetness perception, simultaneously influencing aromatic complexity (47). Venn analysis revealed that hexanoic acid was consistently detected across all samples (Fig. 3B), serving as the direct biochemical precursor for ethyl hexanoate synthesis. The primary production pathway involves synergistic interaction between hexanoate-producing bacteria and methanogens, where ethanol is oxidized to acetate, followed by its reaction with butyrate to form hexanoate (19). As key flavor components in Baijiu, alcohols showed distinct vertical distributions (Fig. 3A): total alcohol content decreased with depth in 3- and 100-year pits, while the 45-year pit displayed a unimodal pattern (increase-peak-decrease). The compounds 3-methyl-2-butanol, n-pentanol, 2-heptanol, and n-hexanol were universally present in all pit mud samples, contributing herbal, cereal-like, and fruity aromas to the Baijiu (48). In summary, the volatile components in pit mud reflect the evolution of microbial ecological functions during the aging process to a certain extent, providing an important basis for elucidating the relationship between pit mud quality and Baijiu flavor.

#### Analysis of key differential compounds in pit mud and their correlation with metal elements

OPLS-DA modeling of volatile compounds across spatiotemporal pit mud gradients demonstrated model reliability (*R*^2^ = 0.578, *Q*^2^ = −0.705) with 200-permutation tests confirming no overfitting (Fig. 3C). The 3D score plot revealed distinct volatile compound profiles among pit mud samples of varying ages and spatial locations, with BD and BM demonstrating clear separation from other groups. Compounds were prioritized based on variable importance in projection (VIP > 1) and odor activity value (OAV > 1; Table 1) (49). Eight key differential compounds were identified: ethyl hexanoate, hexanoic acid, butyl hexanoate, propyl hexanoate, ethyl caprylate, hexyl hexanoate, ethyl heptanoate, and 1-hexanol. These compounds account for the aroma variations observed across different pit ages and spatial layers.

**TABLE 1:** Volatile compounds with VIP > 1 and OAV > 1

Number	Compound	Threshold (µg/L)	VIP value	OVA value								
				AT	AM	AD	BT	BM	BD	CT	CM	CD
3	Ethyl hexanoate	55	3.24	>1	>1	>1	>1	>1	>1	>1	>1	>1
49	Hexanoic acid	2,520	2.75	>1	>1	>1	>1	>1	>1	>1	>1	>1
7	Butyl hexanoate	700	2.23	>1	>1	>1	>1	>1	>1	>1	>1	>1
5	Propyl hexanoate	12,783.77	1.72	>1	>1	>1	>1	>1	>1	>1	>1	>1
8	Ethyl caprylate	13	1.64	>1	>1	>1	>1	>1	>1	>1	>1	>1
13	Hexyl hexanoate	1,890	1.41	>1	>1	>1	>1	>1	>1	>1	>1	>1
42	1-Hexanol	5,370	1.35	>1	>1	>1	>1	>1	>1	>1	>1	>1
6	Ethyl heptanoate	13,153.17	1.21	>1	>1	>1	>1	>1	>1	>1	>1	>1

The concentration of metal ions in Baijiu significantly modulates organic compound formation, ultimately determining the liquor’s flavor profile (50). Metal ions from pit mud migrate into fermented grains during fermentation, with a minor fraction transferring into the distilled liquor (2). Therefore, redundancy analysis was further conducted on the key differential substances and metal elements in the pit mud to investigate the correlation between volatile components and metal elements in the pit mud (Fig. 3F). The study found that organic acids promote the transformation of metal ions from complexed states to free states. Consequently, Fe²^+^ can facilitate the hydrolysis of esters, increasing the production of corresponding acids, which is why it shows a positive correlation with hexanoic acid (39, 51). In addition, potassium (K) exhibited a positive correlation only with 1-hexanol but showed negative correlations with other compounds. In contrast, magnesium (Mg), manganese (Mn), zinc (Zn), and calcium (Ca) were positively correlated with all compounds except hexanoic acid. These findings indicate that metal elements can influence the aroma components of pit mud in different spatial locations.

### Archaeal community composition and diversity in pit mud across different spatial ages

#### Alpha- and beta-diversity of archaea in pit mud

**Fig 4:**
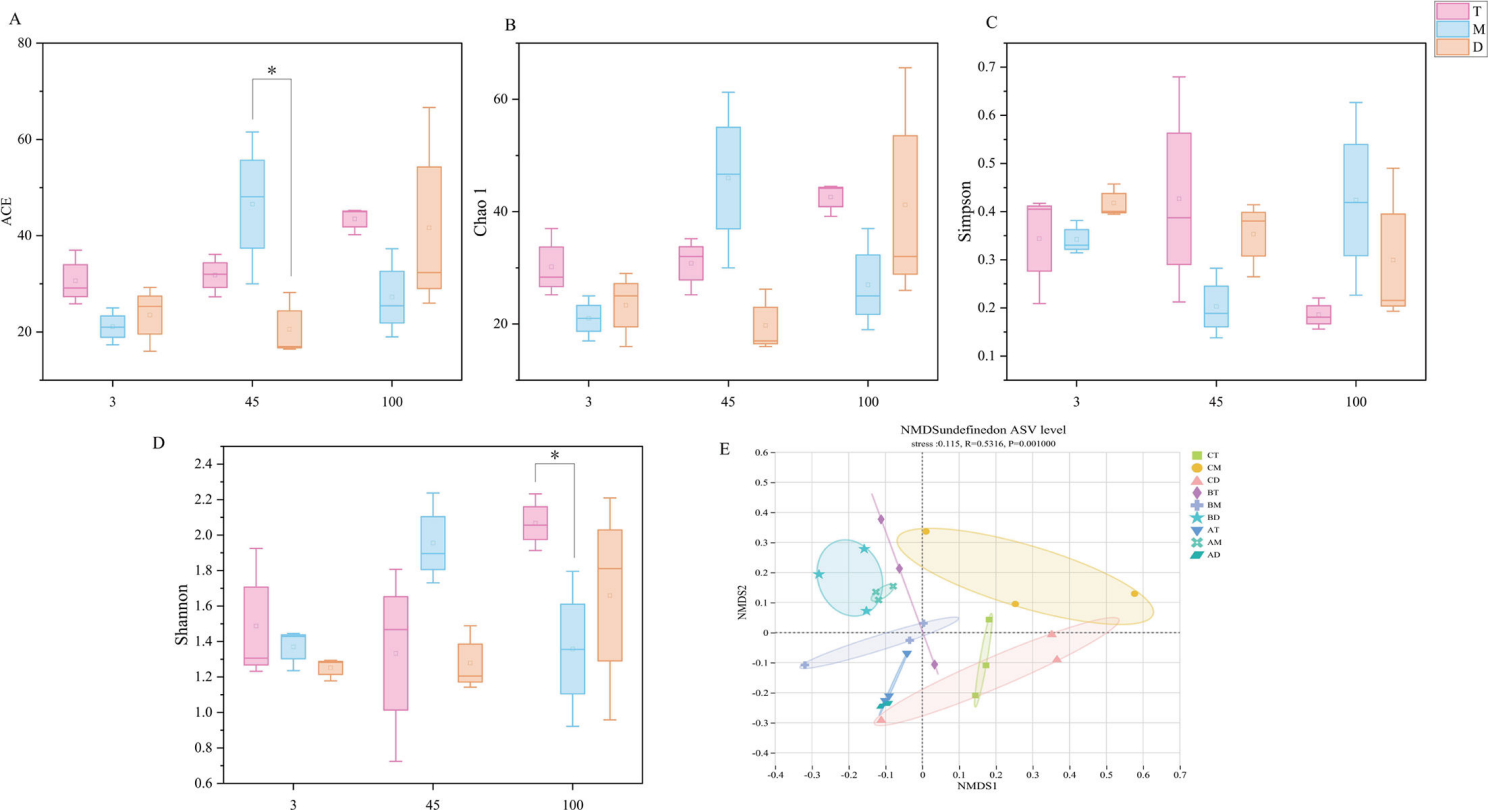
Archaeal α-diversity in pit mud (**A–D**) and non-metric multidimensional scaling (NMDS) analysis of archaeal communities across temporal strata (**E**).

High-throughput sequencing was employed to investigate archaeal communities across vertical profiles of pit mud with different aging periods (3, 45, and 100 years). The archaeal community composition in pit mud was characterized using the ACE and Chao1 indices (representing richness), Shannon and Simpson indices (representing diversity), and the coverage index (representing sequencing depth) (52). Within the same pit, the archaeal species richness (Chao1 and ACE) was lowest in the middle layer for both 3- and 100-year pit muds, while it peaked in the middle layer for the 45-year pit mud. The Shannon and Simpson indices demonstrated an overall decreasing trend in archaeal diversity with increasing pit depth (Fig. 4C and D). Among them, CT exhibited the highest archaeal diversity (2.07 ± 0.99), followed by BM (1.95 ± 0.64). Chen Rangfang et al. discovered through microbial research in pit mud that direct or indirect relationships exist between archaeal communities and volatile compounds (53). Therefore, BM pit mud demonstrated both high richness and diversity of archaea, which may partially account for its greatest variety and abundance of volatile compounds.

NMDS and ADONIS analyses based on Bray-Curtis distance were employed to investigate similarities and differences in archaeal communities of pit mud across temporal gradients (Fig. 4E). The results revealed significant differences in archaeal communities of pit mud across different aging years and spatial positions (*P* = 0.001, *R* = 0.5316) (14). From the perspective of pit age, pit mud samples from identical spatial positions but different aging periods were distributed in distinct quadrants, indicating significant differences in archaeal community composition. Furthermore, the microbial communities in 3- and 45-year pit muds showed greater similarity to each other while being clearly distinguishable from those in 100-year pit muds. From a spatial perspective, CM was distinctly separated from pit mud at other positions, showing significant differentiation from all other samples, followed by CT and CD, which demonstrated relatively higher similarity to each other. Additionally, BT and BD exhibited similar clustering patterns, as did AD and AT. These results demonstrate that both the pit age and spatial positioning significantly influence the similarity and differentiation patterns of archaeal communities.

#### Analysis of archaeal community structure in pit mud across spatiotemporal age

**Fig 5:**
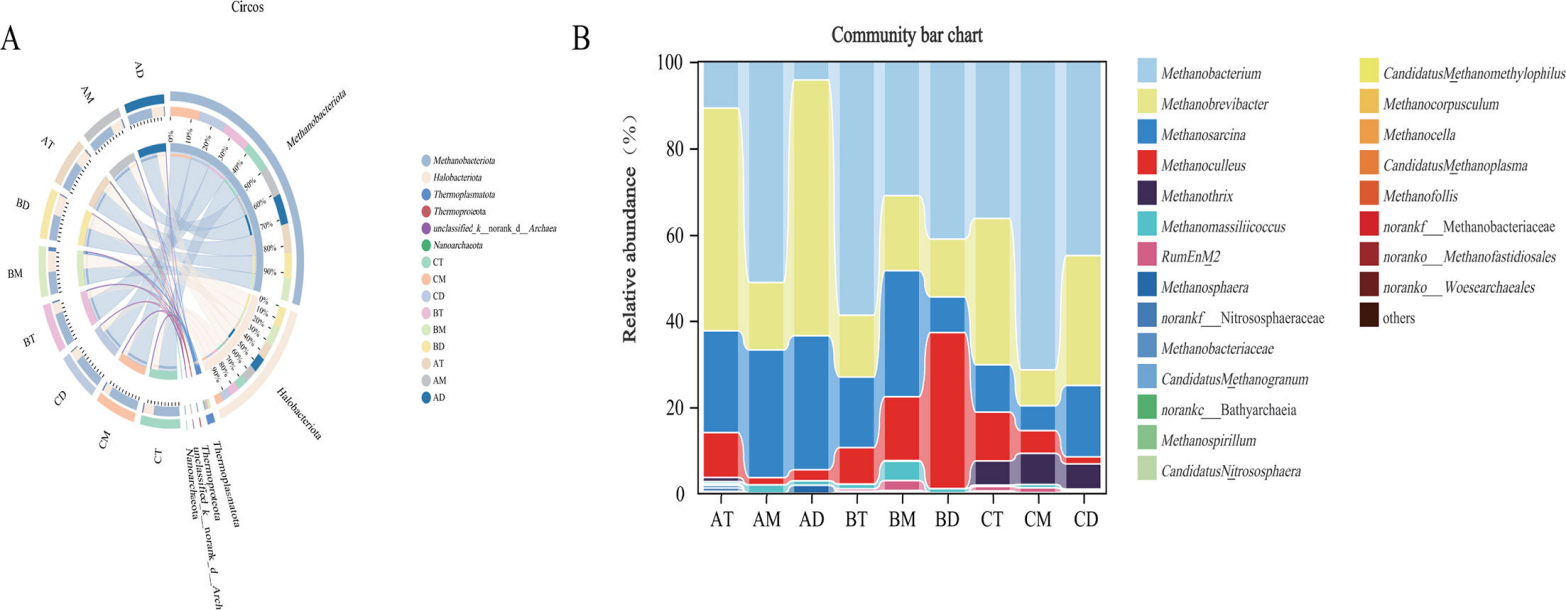
(**A**) Circos plot depicting the composition and relative abundance of archaeal communities in pit mud at the phylum level. (**B**) Bar chart illustrating the taxonomic structure of archaeal communities in pit mud at the genus level.

Archaea serve as essential microbial components in traditional Baijiu fermentation, with characteristically enriched populations observed in mature pit mud ecosystems. Previous studies have revealed that the archaeal community in pit mud is primarily composed of *Euryarchaeota* and *Thaumarchaeota*, with *Euryarchaeota* being the absolutely dominant phylum accounting for over 99% of the relative abundance (19). The reason might be that the climate (such as temperature and humidity) in different production areas affects the fermentation process and metabolite accumulation in the cellar, and the differences in raw materials and brewing techniques result in different microbial growth environments in the cellar mud, thereby forming different archaeal community structures. The results of this study indicate that a total of 23 archaeal genera and 5 archaeal phyla were detected in the pit mud. At the phylum level (Fig. 5A), the main phyla included *Methanobacteriota* (formerly *Euryarchaeota*), *Halobacteriota*, *Thermoplasmatota*, *Thermoproteota*, and *Nanoarchaeota*. Among them, *Methanobacteriota* and *Halobacteriota* accounted for over 90% of all archaeal phyla in the pit mud, representing the absolutely dominant phyla. This finding differs from previous studies. Overall, as the pit age increased, *Methanobacteriota* exhibited the highest abundance in 100-year-old pit mud. However, in pit mud samples from locations BM and BD, its abundance was lower than that in 3-year-old pit mud. In contrast, *Halobacteriota* showed the highest abundance (approximately 44%) in these two locations. *Nanoarchaeota* were exclusively detected in sample AT among all tested samples.

At the genus level (Fig. 5B), all pit mud samples consistently contained five archaeal genera: *Methanobacterium*, *Methanobrevibacter*, *Methanosarcina*, *Methanoculleus*, and *Methanomassiliicoccus*, showing a distinct profile from previous studies (54). Moreover, *Methanothrix* was exclusively detected in 100-year-old pit mud with peak abundance in the middle layer, indicating its specialized adaptation to aged pit ecosystems. The vast majority of archaea in pit mud belong to methanogenic genera, and the abundance of these genera varies with the age of the pit (19, 55). *Methanoculleus* reached its highest abundance in 45-year pit mud, showing an increasing trend with pit depth, with BD accounting for 36.03%. *Methanosarcina*, capable of utilizing both acetate and H_2_, showed higher abundance in new pit muds but exhibited an overall decreasing trend with pit age (55, 56). *Methanobacterium* can utilize acetate, formate, and methanol as substrates, while *Methanosarcina* is capable of simultaneous utilization of CO_2_ with either acetate or methanol (57, 58). Furthermore, *Methanothrix* is an obligate acetoclastic methanogen that converts acetate to methane and carbon dioxide through acetate disproportionation. This metabolic pathway enables *Methanothrix* to thrive in acetate-rich anaerobic environments (59). Methanol, a toxic compound, induces poisoning upon excessive ingestion in alcoholic beverages, with severe outcomes including visual impairment, coma, and fatal consequences (60). Therefore, a higher abundance of methanol-utilizing methanogens may promote methanol reduction in Baijiu, thereby benefiting human health. Furthermore, the syntrophic interaction between methanogens and caproic acid-producing bacteria relieves hydrogen inhibition, facilitating acid accumulation during fermentation and ultimately contributing to the formation of characteristic flavor compounds in strong-aroma Baijiu (61).

### Correlation analysis between archaea and physicochemical/flavor characteristics

**Fig 6:**
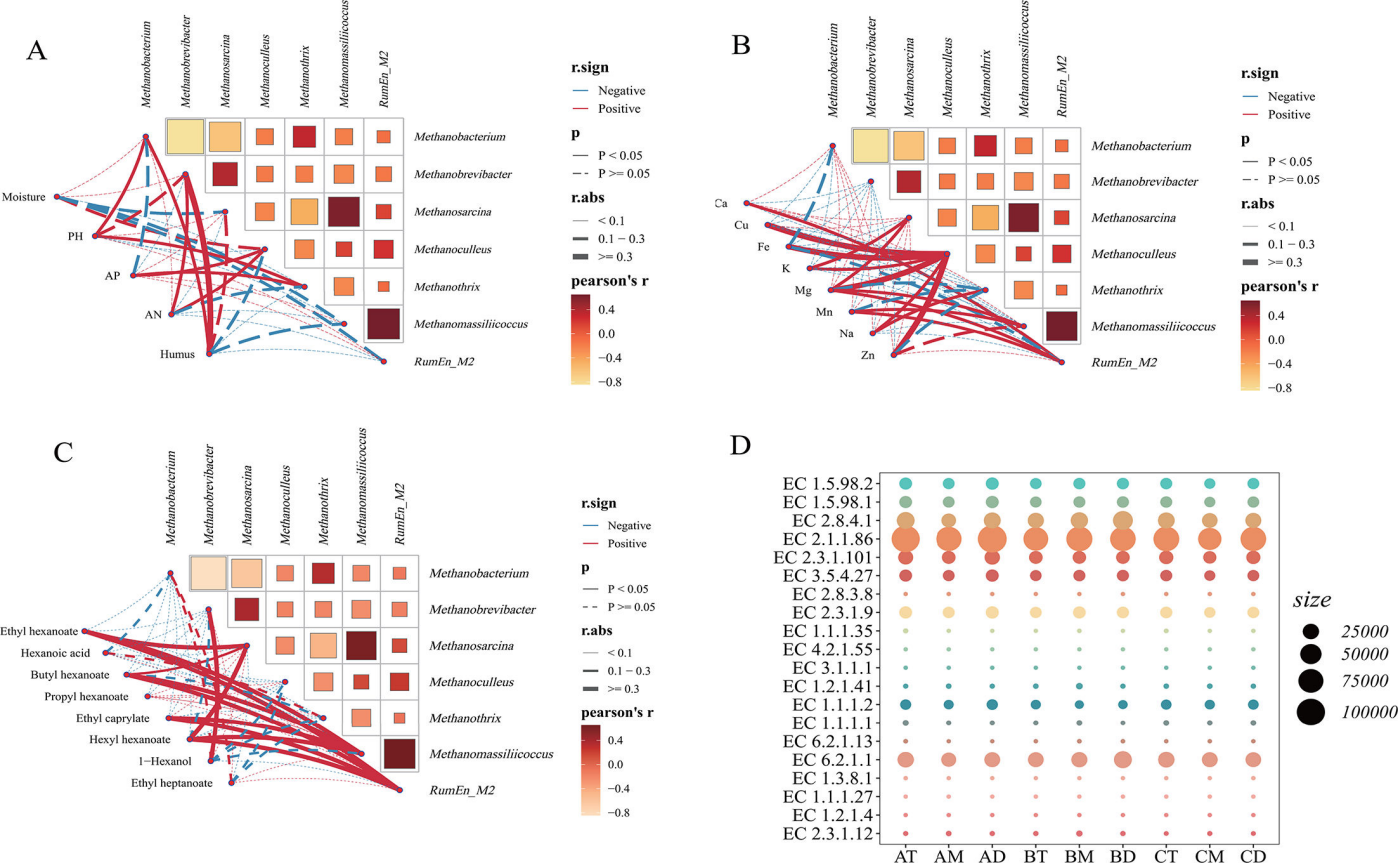
Correlation analysis between physicochemical parameters and dominant archaeal genera. (**A**) Archaea-physicochemical indices. (**B**) Archaea-metal ions. (**C**) Archaea-key flavor compounds. (**D**) Bubble chart of key archaeal metabolic enzymes.

To further investigate the influence of physicochemical factors and metal elements on archaeal communities in pit mud, we evaluated the relationships among eight core archaeal genera, five physicochemical parameters, and eight metal elements based on Pearson correlation coefficients (Fig. 6A and B). The correlation results indicate that *Methanobacterium* has a significant positive correlation with pH and humus, *Methanobrevibacter* has a significant positive correlation with AN and humus, and *Methanoculleus* has a significant positive correlation with AP and AN. This suggests that changes in physical and chemical factors can affect the abundance of different archaeal genera. The methanogenic archaea *Methanobacterium* and *Methanosarcina* serve as reliable biomarkers for pit mud maturation, suggesting that targeted modulation of physicochemical parameters could be employed to optimize pit mud quality (62). Metal elements have been relatively rarely mentioned in existing studies of pit mud archaeal communities. The results show that metals promote most archaea. *Methanoculleus* showed significant positive correlations with all tested metal ions except Ca and Mn, *Methanosarcina* exhibited significant positive correlations with K and Mg, and *RumEn_M2* was significantly positively correlated with Ca, Mg, and Na. Among them, K^+^ and Na^+^ help maintain intracellular-ion homeostasis with the external environment, thereby influencing archaeal community structure (63). In conclusion, pit mud archaeal communities are co-regulated by various physicochemical factors and metal elements, although their precise mechanistic impacts require further investigation.

The microbial community in PM co-metabolizes to form distinct flavor characteristics. Therefore, further correlation analysis between core archaeal genera and key flavor compounds was conducted (Fig. 6C). *Methanobrevibacter* shows a significant positive correlation with 1-hexanol. *Methanosarcina* shows a significant positive correlation with ethyl hexanoate, butyl hexanoate, and hexyl hexanoate. RumEn_M2 shows a significant positive correlation with hexyl hexanoate, ethyl caprylate, butyl hexanoate, and ethyl hexanoate. A single compound (e.g., hexyl hexanoate or butyl hexanoate) can show positive correlations with different archaeal genera, suggesting these flavor compounds may be co-produced through archaeal co-fermentation. This phenomenon likely occurs because the synthesis of such compounds requires multiple enzymes derived from different microbial species (64).

### Functional prediction analysis of archaeal communities in pit mud

**Fig 7:**
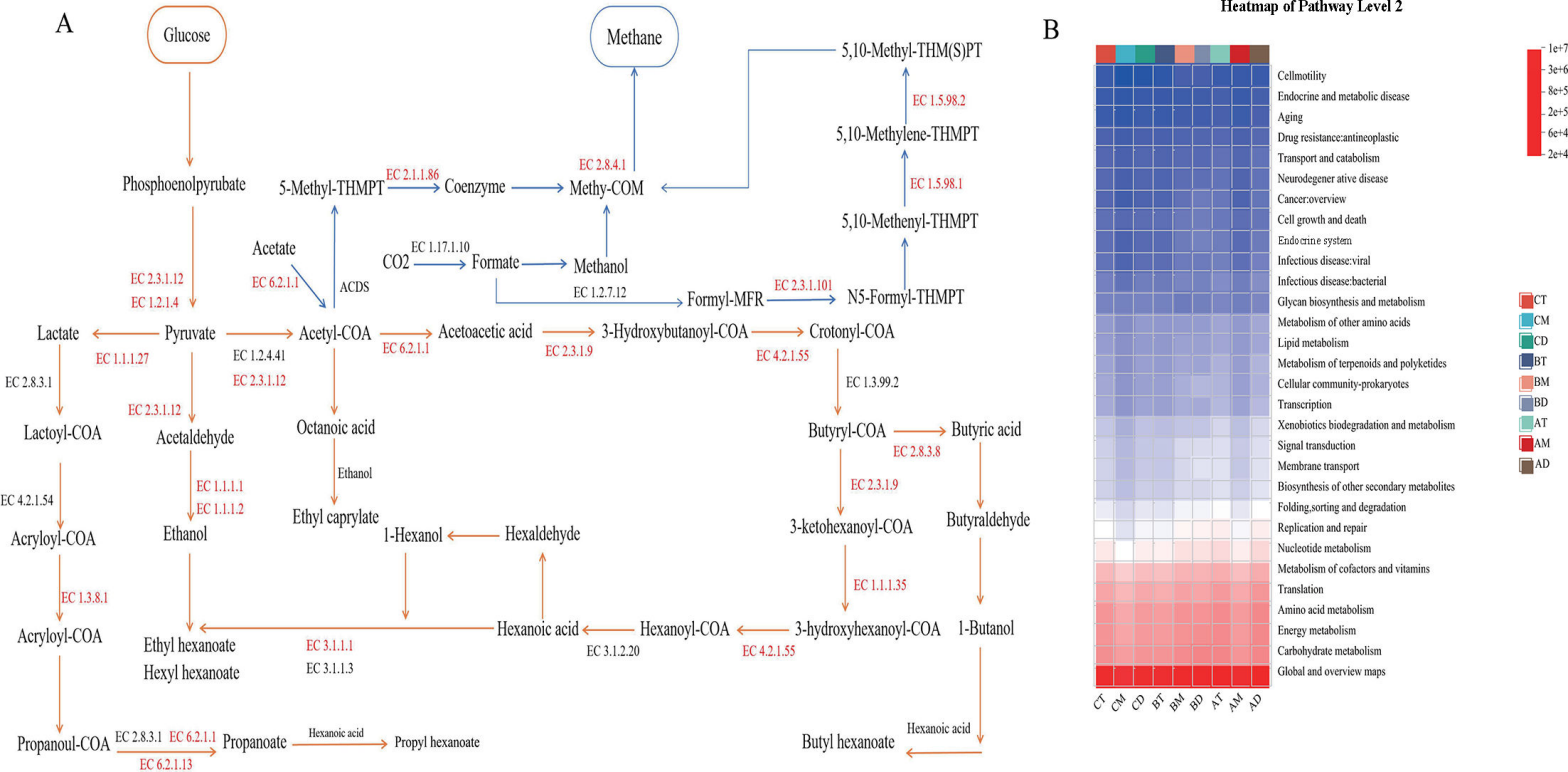
(**A**) Functional profile of pit mud archaeal communities predicted by PICRUSt2 based on 16S rRNA sequencing data. (The functional pathways presented in the text are prediction results only.) (**B**) Heatmap depicting the relative abundance of microbial functional pathways at KEGG level 2 (with rows representing pathway categories, columns representing different pit mud groups).

The heatmap of KEGG level 2 pathway abundance (Fig. 7B) revealed that carbohydrate metabolism exhibited the highest relative abundance among all pathways, excluding global maps and overview maps. The secondary predominant pathways were energy metabolism and amino acid metabolism, indicating that starch and protein degradation serve as the fundamental biochemical basis for flavor compound formation (65). Therefore, to further elucidate the metabolic and functional characteristics of archaeal communities in PM, we investigated the roles of key enzymes involved in carbohydrate hydrolysis, energy metabolism, and the formation of critical flavor compounds across different pit mud samples.

For carbohydrate metabolism, the predominant pathways included glycolysis/gluconeogenesis, pyruvate metabolism, and the citrate cycle (tricarboxylic acid cycle) (66). A total of 18 enzymes were annotated in carbohydrate metabolism, with acetyl-CoA synthetase (EC 6.2.1.1), alcohol dehydrogenase [NADP(+)] (EC 1.1.1.2), and acetyl-CoA acetyltransferase (EC 2.3.1.9) identified as key enzymes (Fig. 6C). However, their abundance showed no clear correlation with either pit age or depth. The results demonstrated that EC 6.2.1.1 was most abundant in BD, while EC 1.1.1.2 and EC 2.3.1.9 showed the highest levels in AD. Notably, EC 1.1.1.2 worked synergistically with alcohol dehydrogenase (EC 1.1.1.1) to facilitate the interconversion between acetaldehyde and ethanol (67). Furthermore, the study revealed that butyrate and hexanoate production is co-regulated through synergistic interactions between bacterial and archaeal communities, whereas methane metabolism is predominantly governed by archaeal communities (41). The functional analysis of energy metabolism primarily focused on methanogenesis, with six annotated enzymes identified as pivotal to the process: methylenetetrahydromethanopterin cyclohydrolase (EC 3.5.4.27), formylmethanofuran-tetrahydromethanopterin N-formyltransferase (EC 2.3.1.101), coenzyme-B sulfoethylthiotransferase (EC 2.8.4.1), methylenetetrahydromethanopterin dehydrogenase (EC 1.5.98.1), and 5,10-methylenetetrahydromethanopterin reductase (EC 1.5.98.1). Methanogenesis typically proceeds through three pathways (Fig. 7A): the CO_2_ reduction pathway, methylotrophic pathway, and acetoclastic pathway, with enzymes associated with the acetoclastic pathway (particularly EC 2.8.4.1) exhibiting the highest abundance, especially in BD samples (41). Acetate serves as a critical metabolic intermediate in methane production, with the acetoclastic methanogenesis pathway contributing approximately two-thirds of naturally occurring methane globally (59). In the acetoclastic methanogenesis pathway, EC 6.2.1.1 plays a pivotal role in catalyzing the conversion of acetate to acetyl-CoA, which is subsequently transformed into the methane precursor 5-methyl-THMPT via the acetyl-CoA decarbonylase/synthase complex. Methane generation is then finalized through sequential reactions mediated by EC 2.8.4.1 (coenzyme-B sulfoethylthiotransferase) and EC 2.1.1.86 (methyl-transferase) (59).

### Conclusion

This study reveals how pit mud influences liquor flavor by analyzing physicochemical properties, archaeal succession, and flavor compounds across different ages (3/45/100 years) and depths. The results demonstrate that both pit age and spatial positioning significantly influence the distribution of metal elements, physicochemical characteristics, and archaeal community structure in pit mud, thereby regulating the synthesis of flavor compounds. The archaeal community was dominated by methanogens, whose abundance exhibited dynamic shifts along the pit-age gradient, reflecting temporal succession patterns in the pit mud microecosystem. Furthermore, functional prediction analysis elucidated the metabolic potential of archaeal communities, providing a theoretical foundation for both pit mud quality assessment and flavor modulation strategies. Comprehensive evaluation reveals superior quality parameters in 45-year-old versus 100-year-old pit mud. This finding suggests that sustained optimization and maintenance are essential for preserving the optimal performance of aging pit mud. In this study, we shifted the research focus from the extensively investigated bacterial communities to the succession of archaeal communities during pit mud aging. By revealing the directional succession patterns of archaea and their correlation with flavor metabolism, this work provides a new direction for optimizing pit mud maintenance and management to enhance the flavor stability of Baijiu. Future studies should integrate multi-omics approaches such as metagenomics, metabolomics, and culturomics to elucidate the functional roles, metabolic interactions, and direct regulatory mechanisms of key archaeal taxa in flavor formation during succession.

## Data Availability

The sequencing data have been deposited in the NCBI SRA with the accession number PRJNA1402108.

## SUPPLEMENTAL MATERIAL

Supplemental material aem.00099-26-s0001.xlsx KEGG prediction analysis data.

ASM does not own the copyrights to Supplemental Material that may be linked to, or accessed through, an article. The authors have granted ASM a non-exclusive, world-wide license to publish the Supplemental Material files. Please contact the corresponding author directly for reuse.
